# Applicability, Validity, and Reliability of the Japanese Version of the Behavioral Pain Scale for Critically Ill Mechanically Ventilated Pediatric Patients: A Prospective Cross-Sectional Observational Study

**DOI:** 10.3390/children13060719

**Published:** 2026-05-22

**Authors:** Mitsuki Ikeda, Haruhiko Hoshino, Yujiro Matsuishi, Misaki Kotani, Shunsuke Kobayashi, Takahiro Kido, Yuki Enomoto, Nobutake Shimojo, Yoshiaki Inoue

**Affiliations:** 1 Department of Emergency and Critical Care Medicine, Faculty of Medicine, University of Tsukuba, Tsukuba 305-8575, Japan; ironman.m16@gmail.com (M.I.);; 2 Department of Nursing, University of Tsukuba Hospital, Tsukuba 305-8576, Japan; 3 Adult Nursing (Acute Care) Department of Nursing, Faculty of Medical Technology, Teikyo University, Tokyo 192-0395, Japan; 4 Department of Nursing Science and Engineering, Women’s Health Nursing and Midwifery, Faculty of Medicine, University of Tsukuba, Tsukuba 305-8575, Japan; 5 Department of Child Health, Institute of Medicine, University of Tsukuba, Tsukuba 305-8576, Japan; 6 Department of Pediatrics, University of Tsukuba Hospital, Tsukuba 305-8576, Japan

**Keywords:** Behavioral Pain Scale, pediatric intensive care unit, pain assessment, psychometrics, validity, reliability, Japan, sedation, mechanically ventilated

## Abstract

**Highlights:**

**What are the main findings?**

- The Japanese version of the Behavioral Pain Scale (BPS) demonstrated acceptable validity and interrater reliability in mechanically ventilated pediatric patients.
- Deep sedation (median RASS score ≤ −4) caused a pronounced floor effect, significantly masking behavioral pain responses even during painful procedures.

**What is the implication of the main finding?**

- Future research is needed to investigate whether the BPS, with adequate training, can serve as a common multidisciplinary language in mixed adult–pediatric intensive care units.
- Clinicians must carefully interpret low BPS scores in deeply sedated children as they may reflect sedation-induced masking rather than true analgesia.

**Abstract:**

**Background:** Pain assessment in critically ill, mechanically ventilated pediatric patients is highly complex owing to communication barriers and the frequent use of sedation. A standardized, rapid, and objective tool such as the Behavioral Pain Scale (BPS) is urgently needed in Japanese pediatric intensive care units (PICUs), particularly in mixed adult–pediatric settings, to ensure consistent, multidisciplinary assessment. This study aimed to evaluate the clinical applicability, validity, and reliability of the Japanese version of the BPS in critically ill mechanically ventilated pediatric patients. **Methods:** This single-center, prospective cross-sectional observational study was conducted between October 2021 and March 2023. The final analysis included 70 observations from 37 pediatric patients who needed mechanical ventilation (MV). Concurrent and convergent validity were assessed using Spearman’s rank correlation coefficients (ρ) between the BPS; the Face, Legs, Activity, Cry, Consolability (FLACC) scale; and the COMFORT-Behavior (COMFORT-B) scale. Interrater reliability was evaluated using intraclass correlation coefficients (ICCs) and weighted kappa values among the three independent observers. The sample size (52 observations) was calculated based on the kappa coefficient estimation. The impact of sedation depth (assessed using the Richmond Agitation–Sedation Scale [RASS]) and the observers’ prior clinical experience with the evaluations were also analyzed. **Results:** Concurrent and convergent validity were high, showing strong correlations with the FLACC (ρ = 0.49–0.91) and COMFORT-B (ρ = 0.69–0.87) scales. The total BPS score showed moderate interrater reliability (ICC = 0.66, 95% CI = 0.55–0.76; weighted κ = 0.63–0.71). However, deep sedation (defined as a median RASS score ≤ −4 across observers), present in 68.6% of the observations, caused a pronounced floor effect that suppressed behavioral responses, even during painful procedures. Consequently, the reliability of fine motor subscales like “upper limb movement” (κ = 0.08) was slight and for “facial expression” (κ = 0.38), it was fair. Furthermore, the correlation strength with the FLACC scale varied significantly with observer experience, with the strongest correlation (ρ = 0.91) achieved by the observer with extensive adult ICU experience. **Conclusions:** As an initial validation, the Japanese version of the BPS has demonstrated acceptable validity and moderate reliability in mechanically ventilated pediatric patients. However, its clinical application requires careful interpretation because of the pronounced floor effect under deep sedation. Furthermore, accurate assessment depends heavily on specific training and familiarity with the adult-derived scale. With adequate training, the BPS has the potential to serve as an alternative tool and a valuable common multidisciplinary language in mixed intensive care settings. Future research should investigate whether implementing this tool improves multidisciplinary communication and clinical outcomes.

## 1. Introduction

Pain management in critically ill children admitted to pediatric intensive care units (PICUs) is highly complex. Owing to developmental differences and the frequent use of mechanical ventilation (MV) or sedation, verbal communication is often impossible [1]. Inadequate pain management has been linked to prolonged MV, extended intensive care unit (ICU) stay, and drug dependence due to oversedation [2,3]. Therefore, standardized behavioral observation tools are essential to minimize interrater variability and accurately assess pain in noncommunicative pediatric patients [4,5,6].

Currently, objective behavioral pain assessment tools recommended internationally for PICUs include the Face, Legs, Activity, Cry, Consolability (FLACC) and COMFORT-Behavior (COMFORT-B) scales [7,8,9,10,11]. In particular, there is extensive evidence of the validity and reliability of the FLACC scale in pediatric populations and it is widely regarded as the reference standard for behavioral pain assessment [12].

While other tools, such as the Critical-Care Pain Observation Tool (CPOT), exist, they may require slightly longer assessment times than simpler behavioral scales [13].

The Behavioral Pain Scale (BPS) is a well-established, rapid, and objective pain assessment tool originally developed for adult ICU patients requiring sedation and MV [14]. It consists of three simple subscales (facial expression, upper limb movement, and compliance with ventilation) and has been extensively validated in adult populations [14,15,16,17,18,19,20]. In Japan, the BPS is strongly recommended by the Japanese Guidelines for the Management of Pain, Agitation, and Delirium (J-PAD) and is the most widely used objective pain scale for nonverbal adult patients [21].

Recently, several studies outside Japan have demonstrated the reliability and validity of the BPS in pediatric critical care settings, Specifically, the BPS was validated by Sulla et al. in infants (all under 1 year of age), Tamvaki et al. in children aged 1 month to 15 years (mean age: 4.10 ± 5.30 years), and Guasconi et al. in neonates and children aged 0 to 15 years [22,23,24], all reporting high internal consistency and strong correlations with pediatric-specific scales such as FLACC and COMFORT-B. These findings suggest that the BPS can be a highly practical tool for children.

Validating the BPS for pediatric use is particularly important in Japan, where pediatric and adult patients are frequently managed in the same general ICU, or by multidisciplinary staff rotating between pediatric and adult care. A common age-transcending assessment tool with a brief observation time can significantly reduce staff workloads, promote consistency, and foster a shared understanding of pain assessment among multidisciplinary teams.

However, the validity, reliability, and clinical applicability of the Japanese version of the BPS have not been evaluated in a PICU setting. Addressing this knowledge gap is essential to establish standardized evidence-based pain management protocols. Therefore, this study aimed to evaluate the validity, reliability, and clinical applicability of the Japanese version of the BPS among critically ill, mechanically ventilated pediatric patients. We hypothesized a priori that the Japanese version of the BPS would demonstrate acceptable concurrent and convergent validity (Spearman’s rho > 0.50) [25] and moderate to good interrater reliability (intraclass correlation coefficient (ICC) > 0.60) [26].

## 2. Materials and Methods

### 2.1. Study Design and Setting

This prospective cross-sectional observational study was conducted in the PICU of the University of Tsukuba Hospital, a tertiary university hospital in Japan with approximately 800 inpatient beds. The PICU consisted of six open beds and two private isolation rooms (eight beds in total). The unit operates under a semi-closed management system in which pediatric intensivists and attending subspecialty physicians collaborate to provide intensive care to critically ill children. Patients admitted to the PICU included those who had undergone elective surgery, those with acute exacerbations of endogenous diseases, and those admitted to the emergency department from outside hospitals.

### 2.2. Participants

Children younger than 18 years who stayed in the PICU for at least 24 h and required MV between October 2021 and March 2023 were eligible. The exclusion criteria were as follows: PICU stay < 24 h, age ≥ 18 years, did not require MV, had a neuromuscular disorder, and was receiving neuromuscular blocking agents during observation. To minimize the bias from repeated measurements, a maximum of five assessments per patient were allowed. This study was approved by the Institutional Review Board of the University of Tsukuba Hospital (H28-085). Informed consent was waived due to the observational nature of the study, which utilized clinical data obtained during routine practice without any additional interventions. The waiver of written informed consent and the use of an opt-out method were formally approved by the Ethics Committee.

### 2.3. Sample Size

Convenience sampling was used to recruit patients from the PICU who met the eligibility criteria during the study period. The sample size was estimated based on previous studies and the confidence interval estimation of the weighted kappa coefficient (κ). Assuming κ = 0.7 with a standard deviation (SD) of 0.5, the required sample size was calculated to be 52 observations The expected SD of 0.5 was based on conservative estimates from prior behavioral scale validations. To allow for potential missing data, we ultimately included 70 observations [27,28,29]. All calculations were performed using EZR software (version 4.0; Jichi Medical University, Saitama, Japan) [30].

### 2.4. Measurement Tools

In addition to the BPS, this study employed the FLACC [12] and COMFORT-B [31] scales. Furthermore, the Richmond Agitation–Sedation Scale (RASS) [32] was used to collect data on the depth of sedation as part of the patients’ background characteristics. The details of each scale used in this study are described below.

#### 2.4.1. Behavioral Pain Scale (BPS)

The BPS consists of three domains: facial expression, upper limb movement, and compliance with MV (or vocalization in non-intubated patients). Each item is rated on a scale of 1 to 4, yielding a total score ranging from 3 to 12. A score of 3 indicates no pain, 4–5 indicates mild to moderate pain, 6–11 indicates severe pain, and 12 indicates the most intense pain imaginable. The Japanese version of the BPS, translated and validated by the Japanese Society of Intensive Care Medicine (J-PAD) Guidelines Committee, was used in this study [6].

#### 2.4.2. Face, Legs, Activity, Cry, Consolability (FLACC) Scale

The FLACC scale consists of five domains (facial expression, leg movement, activity, crying, and consolability), each rated on a 0–2 scale, resulting in a total score of 0–10. A score of 0 indicates no pain, 1–3 indicates mild discomfort, 4–5 indicates moderate discomfort/pain, and 6–10 indicates severe pain; a score ≥4 was considered indicative of pain [12]. In this study, the FLACC scale was adopted as the reference standard for evaluating the concurrent validity of the BPS owing to its robust psychometric properties in pediatric intensive care. Our group developed and validated the Japanese version [33].

#### 2.4.3. COMFORT Behavior (COMFORT-B) Scale

The COMFORT-B scale comprises six domains: alertness, calmness/agitation, respiratory response or crying, body movement, muscle tone, and facial tension. Each item is scored on a scale of 1 to 5, yielding a total score of 6 to 30 [31]. Scores of 6–13 indicate no distress, 14–21 indicate moderate distress, and ≥22 indicate severe distress; a score ≥17 was considered to indicate pain [1].

#### 2.4.4. Richmond Agitation–Sedation Scale (RASS)

The RASS assesses sedation depth on a 10-point scale ranging from −5 (unarousable) to +4 (combative). Deep sedation was defined as a median RASS score of ≤−4 across the three observers [32]. The Japanese version has been previously developed and validated by our group [34]. Recent studies have validated the use of the RASS in pediatric critical care settings [35].

### 2.5. Evaluation and Collection of Data

Three evaluators participated in the study: the principal investigator (Observer A) and two PICU nurses (Observers B and C). Observers A and B had >5 years of PICU experience, whereas Observer C had >5 years of adult ICU experience and 3–5 years of PICU experience. Observer C was familiar with the BPS and FLACC scale because of prior clinical use. One month before data collection, a training session was conducted by the principal investigator covering the details and rating procedures of the BPS, FLACC, and COMFORT-B scales. A one-month pilot test (September 2021) was conducted to refine the observation techniques and ensure interrater consistency. During the study, the three observers evaluated each patient simultaneously and independently once weekly at the same time point without communication during the assessment.

Specific clinical data were recorded at the time of each observation, including sex, age, body weight, disease category, pediatric SOFA (pSOFA) score, use of sedatives and analgesics, depth of sedation (RASS), and presence of painful procedures. Demographic and baseline clinical data, including sex, disease category, admission route, length of stay, length of MV, developmental delay, trisomy status, and pediatric risk of mortality III (PRISM III) score, were extracted from the electronic medical records at PICU admission.

### 2.6. Statistical Analysis

The patient characteristics are summarized as frequencies, means, and standard deviations. The relationships between BPS score and the presence of painful procedures or deep sedation (median RASS score ≤ −4 across observers) were analyzed using the Mann–Whitney U test.

The validity of the BPS was examined by assessing the concurrent validity (correlation with FLACC scale) and convergent validity, a form of construct validity (correlation with COMFORT-B scale). For concurrent validity, Spearman’s rank correlation coefficient (ρ) between the BPS and FLACC scale was calculated. For convergent validity, correlations between the BPS and COMFORT-B scale were computed [22,23,24,36].

The reliability of the BPS was examined through interrater reliability, using both ICC and weighted Cohen’s kappa coefficients (κ). For interrater reliability, the ICC was calculated for overall agreement, and weighted Cohen’s κ was computed for the total and subscale scores. The ICC (two-way random-effects model, absolute agreement, single measures [ICC(2,1)]) was calculated. Interpretation thresholds followed Koo and Li (2016) for ICC (poor: ≤0.50; moderate: 0.50–0.75; good: 0.75–0.90; excellent: >0.90) [26], and Landis and Koch (1977) for κ (<0.00, poor; 0.00–0.20, slight; 0.21–0.40, fair; 0.41–0.60, moderate; 0.61–0.80, substantial; 0.81–1.00, almost perfect agreement) [37]. Correlation strengths were interpreted as follows: 0.0–0.2, negligible; 0.2–0.4, weak; 0.4–0.7, moderate; 0.7–0.9, strong; and 0.9–1.0, very strong [25].

### 2.7. Sensitivity Analysis

To account for the potential floor effect caused by deep sedation-masking behavioral expressions, we conducted a sensitivity analysis. We excluded patients under deep sedation (defined as median RASS score ≤ −4 across observers) and compared BPS scores between the resting state and during the painful procedures in the remaining subgroup of patients under light to moderate sedation (RASS score of 0–3). We used the Mann–Whitney U test to calculate the effect size (r). To evaluate the scoring tendencies of each pain assessment tool, we examined the frequency distributions of the BPS, FLACC, and COMFORT-B scores for each independent observer. Distributions were visually assessed using bar charts and quantitatively summarized in frequency tables.

To compare the strengths of correlations between observers, we used the Fisher z-transformation. All analyses were performed using R version 4.5.1 and RStudio version 2025.09.2+418, with a two-tailed *p*-value < 0.05 considered statistically significant.

## 3. Results

### 3.1. Participant Characteristics

**Figure 1:**
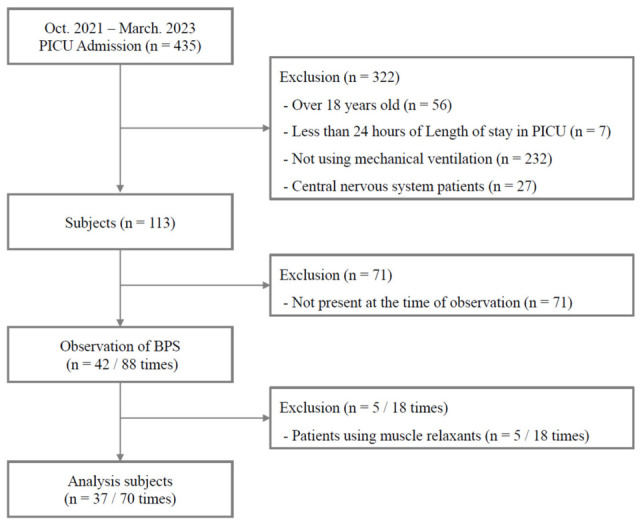
Flowchart for selecting study patients. Abbreviations: BPS—Behavioral Pain Scale; PICU—Pediatric Intensive Care Unit.

Among the 435 patients admitted to the PICU during the study period, 113 met the inclusion criteria after applying the exclusion conditions. Of these, 42 patients (88 observations) underwent BPS assessment. Five patients (18 observations) who were receiving neuromuscular blocking agents at the time of assessment were excluded, leaving 37 patients with 70 observations for the final analysis (Figure 1). The clinical characteristics of the 70 observation points are summarized in Table 1, and the baseline characteristics of the 37 patients at the time of PICU admission are shown in Table S1. Twenty-six patients (70.3%) were male, with a mean age of 1.4 ± 3.9 years at admission (mean: 19.3 ± 47.1 months). The mean length of PICU stay was 30.2 ± 45.6 days. The most common diagnosis was congenital heart disease (n = 30, 81.1%). Of the 70 assessments, 50 (71.4%) were in male patients, and the mean age was 1.7 ± 4.6 years at the time of observation (mean: 23.1 ± 55.0 months). Interventions for pain were performed in 15 (21.4%) patients.

### 3.2. Associations Between BPS Score, Painful Procedure and Deep Sedation

There was no significant difference in the BPS scores between patients who did and did not undergo a painful procedure at the time of observation (Figure S1). The interrater reliability for the RASS scores among the three observers was excellent (ICC = 0.83 [95% CI: 0.76–0.88]). Deep sedation (defined as a median RASS score of <=−4 across the three observers) was observed in 48 (68.6%) observations, which showed significantly lower BPS scores compared with those who were not deeply sedated (22 observations, 31.4%) (*p* < 0.05, Figure S2).

In the sensitivity analysis limited to patients with light to moderate sedation (RASS score ≥ −3), we observed no significant increases in BPS scores during painful interventions across all three observers. However, this subgroup analysis (N = 57) was underpowered, and the absence of statistical significance should not be interpreted as an absence of a true clinical effect. Notably, Observer C showed a trend toward higher scores during interventions (*p* = 0.08, r = 0.24). The effect sizes of the interventions in this subgroup were consistently small (Observer A: r = 0.13; Observer B: r = 0.07; Observer C: r = 0.24) (Table S2).

### 3.3. The Frequency Distributions of Assessed Scores

The frequency distributions of the BPS, FLACC, and COMFORT-B scores evaluated by each observer are shown in Figure S3. Notably, BPS scores exhibited a pronounced floor effect in this deeply sedated cohort. An absolute minimum score of 3 accounted for the vast majority of all observations across the raters (Observer A: 81.4%; Observer B: 75.7%; Observer C: 84.3%). In contrast, while the FLACC and COMFORT-B scores tended toward lower values, they demonstrated relatively wider distributions, indicating some behavioral variability.

### 3.4. Validity

**Figure 2:**
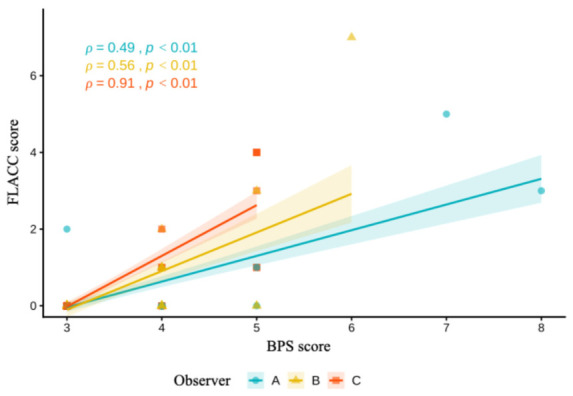
Scatter plots for Spearman’s rho correlation between BPS and FLACC scores: concurrent validity. Abbreviations: BPS—Behavioral Pain Scale; FLACC—Face, Legs, Activity, Crying, Consolability scale. Notes: For Spearman’s rho correlation coefficients between the BPS and FLACC scores for each observer, we plotted a scatter diagram showing the correlation between the BPS and FLACC scores.

The correlations between the BPS and the FLACC scores were as follows: for Observer A, ρ = 0.49 (95% CI: 0.286–0.649, *p* < 0.01); for Observer B, ρ = 0.56 (95% CI: 0.374–0.703, *p* < 0.01); and for Observer C, ρ = 0.91 (95% CI: 0.853–0.941, *p* < 0.01). They demonstrated moderate to strong correlations between the two scales and high concurrent validity (Figure 2). A formal comparison using the Fisher z-transformation confirmed that the correlation for Observer C was significantly stronger than those for Observers A and B (*p* < 0.001).

**Figure 3:**
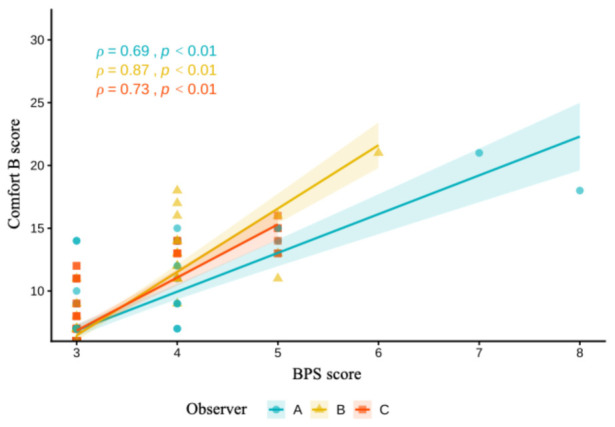
Scatter plots for Spearman’s rho correlation between BPS and COMFORT-B scores: convergent validity. Abbreviations: BPS—Behavioral Pain Scale; COMFORT-B—COMFORT Behavior Scale. Notes: For Spearman’s rho correlation coefficients between BPS and COMFORT-B scores for each observer (A, B and C), we plotted a scatter diagram showing the correlation between BPS and COMFORT-B scores.

Similarly, correlations between the BPS and the COMFORT-B scale were as follows: for Observer A, ρ = 0.69 (95% CI: 0.54–0.80, *p* < 0.01); for Observer B, ρ = 0.87 (95% CI: 0.80–0.92, *p* < 0.01); and for Observer C, ρ = 0.73 (95% CI: 0.59–0.82, *p* < 0.01). This result indicates high convergent validity for the Japanese version of the BPS (Figure 3).

### 3.5. Validity and Impact of Observer Experience

The concurrent validity (compared with FLACC scale) and convergent validity (compared with COMFORT-B scale) of the BPS were further evaluated. Notably, the strength of the correlations varied depending on the clinical background of the observer. For the FLACC scale, Observer C (who had extensive adult ICU experience) demonstrated a remarkably strong positive correlation (ρ = 0.91, *p* < 0.01), whereas Observers A and B (who had less BPS experience) showed a moderate correlation (ρ = 0.53, *p* < 0.01). Conversely, for the COMFORT-B scale, Observers A and B demonstrated a slightly stronger correlation (ρ = 0.78, *p* < 0.01) compared to Observer C (ρ = 0.73, *p* < 0.01). The grouped scatter plots are shown in Figure S4.

### 3.6. Reliability

The interrater reliability results for the overall BPS and its subscale scores are presented in Table 2. The intraclass correlation coefficient (ICC) for the total BPS score among all raters was 0.66 (95% CI = 0.55–0.76; *p* < 0.01), indicating moderate agreement according to the criteria of Koo and Li. The ICCs for each pair of raters ranged from 0.60 to 0.72, also demonstrating moderate agreement.

The weighted Cohen’s kappa coefficients (κ) for the total BPS score were as follows: for Observers A–B, κ = 0.69 (95% CI = 0.50–0.90; *p* < 0.01); for Observers B–C, κ = 0.71 (95% CI = 0.48–0.86; *p* < 0.01); and for Observers A–C, κ = 0.63 (95% CI = 0.28–0.81; *p* < 0.01). These results indicate moderate to substantial agreement among raters. For the subscales, the weighted κ coefficients for compliance with MV were as follows: for Observers A–B, κ = 0.65 (95% CI = 0.51–0.79; *p* < 0.01); for Observers B–C, κ = 0.68 (95% CI = 0.48–0.85; *p* < 0.01); and for Observers A–C, κ = 0.51 (95% CI = 0.23–0.68; *p* < 0.01). These results indicate moderate to substantial agreement. In contrast, the weighted κ coefficient for upper limb movement among all observers was 0.08 (95% CI = −0.03–0.24; *p* = 0.17), indicating poor agreement. For facial expression, the weighted κ coefficient was 0.38 (95% CI = −0.03–0.69; *p* < 0.01), reflecting fair agreement among raters. Overall, these results suggest that the Japanese version of the BPS demonstrates a moderately high reliability for assessing pain in critically ill pediatric patients in the PICU.

## 4. Discussion

This study evaluated the validity, reliability, and clinical applicability of the Japanese version of the BPS in critically ill mechanically ventilated pediatric patients in a PICU setting. Overall, the results demonstrate that the Japanese version of the BPS has acceptable validity and reliability, although its performance is significantly influenced by the depth of sedation and the observer’s clinical experience. To the best of our knowledge, this is the first study in Japan to validate the BPS for pediatric intensive care patients.

To evaluate the concurrent and convergent validity, we compared the BPS with two well-validated pediatric pain scales, the FLACC scale [2,38] and the COMFORT-Behavior (COMFORT-B) scale [8,9,10,11,31], both of which encompass the three behavioral domains included in the BPS [22]. The BPS demonstrated moderate to strong correlations with the FLACC scale (ρ = 0.49–0.91, *p* < 0.01) and strong correlations with the COMFORT-B scale (ρ = 0.69–0.87, *p* < 0.01) (Figure 2 and Figure 3), demonstrating a high convergent validity for the Japanese version of the BPS, consistent with earlier studies [22,23,24].

Regarding reliability, the overall ICC for the total BPS score was 0.66, indicating moderate agreement, and the weighted κ coefficients between observer pairs ranged from 0.63 to 0.71, reflecting moderate to substantial agreement (Table 2). These findings are comparable with those of previous pediatric reports (ICC ranging from 0.88 to 0.96, and κ ranging from 0.60 to 0.98 in Sulla et al., Tamvaki et al., and Guasconi et al.) [22,23,24], suggesting that the Japanese version of the BPS has clinically acceptable reliability. However, when focusing on subscales, the “compliance with ventilation” item showed moderate to substantial agreement (κ = 0.51–0.68), whereas “upper limb movement” (κ = 0.08, *p* = 0.17) and “facial expression” (κ = 0.38, *p* < 0.01) demonstrated only poor to fair agreement (Table 2). This reduced reliability likely reflects the younger patient population in this study (mean age: 1.7 years) and the high proportion of patients under deep sedation (68.6%). Under deep sedation, fine motor or facial expressions are frequently suppressed by sedative agents, making it difficult for multiple observers to interpret subtle behavioral cues consistently.

The suppressive effects of sedation also strongly affected clinical evaluations. Our findings showed no significant increase in BPS scores during painful procedures. Consistent with previous validation studies, we excluded patients who had received neuromuscular blocking agents. However, we intentionally included deeply sedated patients (RASS score of −5) in our observational cohort to reflect the real-world clinical practice in our PICU, where pain assessment (e.g., currently using the FLACC scale [2,38]) is routinely performed as a standard protocol, regardless of sedation depth. The resulting floor effect observed in deeply sedated patients suggests an important clinical consideration: a low behavioral pain score in a deeply sedated child may primarily reflect the masking of behavioral expressions by sedatives, rather than strictly guaranteeing a completely “pain-free” state or adequate analgesia. This failure to detect procedural pain under deep sedation brings into question the fundamental concurrent validity and sensitivity of the scale in this specific population. Cade et al. previously reported that the influence of sedation on pain assessment scales in critically ill patients could not be ruled out [18]. Our findings further support the idea that sedation depth significantly affects behavioral pain assessment in pediatric patients. Therefore, medical care providers should carefully interpret BPS scores under deep sedation and recognize the inherent limitations of behavioral pain scales in these contexts.

Furthermore, this pronounced floor effect should only be interpreted within the specific clinical context of our institution. To date, only a few studies have examined the validity and reliability of the BPS in pediatric settings [22,23,24]. Compared with the cohort reported by Tamvaki et al. [23] (mean age: 4.10 ± 5.30 years; PICU stay: 5.20 ± 7.15 days), our cohort was notably younger (mean age: 1.4 ± 3.9 years) and had a substantially longer PICU stay (30.2 ± 45.6 days). This reflects our PICU’s semi-closed system, which frequently manages high-acuity, long-term pediatric patients who remain under deep sedation for extended periods during the acute phase to ensure safety and strict physiological stability. Unlike modern adult intensive care protocols that favor light sedation and early extubation, this deep sedation strategy is intentionally implemented for critically ill children. Given that the duration of MV in our cohort was comparable to that in previous pediatric reports, we believe that this reflects appropriate and deliberate sedation/analgesia management, rather than unintended oversedation.

Regarding the impact of observer experience on BPS evaluation, using observers with varying levels of clinical experience provided valuable insights into the application of pain assessment tools in the PICU. Observer C, who had approximately six years of adult ICU experience prior to working for three years in the PICU, was highly proficient in using the BPS. This background likely facilitated a more accurate integration of the BPS with the FLACC scale—which shares similar behavioral evaluation components (e.g., facial expression and body movement)—resulting in a remarkably strong correlation (ρ = 0.91). However, the large difference in correlations between observers (0.42) represents a significant threat to the validity and reliability of the scale itself, not merely a training issue. While this finding is limited by the small number of observers, it suggests that extensive experience with the BPS in adult settings could facilitate its application in pediatric settings. In contrast, Observers A and B, who primarily had pediatric experience and were less familiar with the BPS, demonstrated lower agreement between BPS and FLACC scores. This potentially highlights that the BPS, a tool originally developed for adults, may benefit from specific training and familiarity when effectively applied to pediatric patients.

Interestingly, this trend was not observed with the COMFORT-B scale [8,9,10,11,31], where Observers A and B showed slightly stronger correlations than Observer C. Because the COMFORT-B scale evaluates broader aspects of distress and sedation (e.g., alertness and calmness/agitation) rather than just acute pain behaviors, the extensive pediatric clinical experience of Observers A and B may have been advantageous in interpreting these unique pediatric responses. Overall, these findings highlight that while mechanically ventilated pediatric patients share some pain responses with adults, their distinct developmental characteristics make the direct application of adult scales challenging. Therefore, to ensure accurate pain assessment, it is highly desirable to use pediatric-specific tools or develop a modified pediatric-specific version of the BPS.

## 5. Limitations

This study has several limitations. First, this was a single-center study with a relatively small sample size and used convenience sampling, which substantially limits the external validity and generalizability of our findings to other PICU settings across the national healthcare system. Our cohort was heavily dependent on deep sedation, with 90.0% of patients receiving continuous fentanyl, which could have pharmacologically blunted breakthrough pain responses. Future large-scale, multicenter studies should employ a prospective design with stratified sampling by sedation depth to ensure a more balanced evaluation.

Second, our observational design was not strictly event-driven, meaning that the evaluations were not precisely synchronized with the peak acute pain. Moreover, the observed interventions were routine clinical procedures (e.g., vital sign measurements) that might not have induced severe pain. While the overall 70 observations provided sufficient statistical power to assess the primary correlations and overall reliability, the small sample size severely limited the statistical confidence of our subgroup sensitivity analyses. For example, the analysis excluding deeply sedated patients yielded no significant differences, likely because the sample size in this subgroup (n = 4–7 in the intervention group) was insufficient to provide adequate statistical power.

Third, evaluations were conducted by only three observers, one of whom was the principal investigator, which introduces a potential risk of observation bias. Additionally, our observer training was not based on a standardized curriculum.

Fourth, because verbal communication is impossible in this mechanically ventilated population, no true gold standard for pain (such as patient self-report) was available; using the FLACC scale as a behavioral proxy limits the assessment to concurrent validity rather than strict criterion validity. Furthermore, the adult-derived BPS cutoff of ≥5 has not been rigorously validated for pediatric populations.

Finally, given the exploratory nature of this study and the limited number of repeated observations per patient, we treated the observations independently; this lack of adjustment for patient-level clustering may have inflated Type I error rates.

## 6. Conclusions

In conclusion, as an initial validation study, our findings indicate that the Japanese version of the BPS demonstrated acceptable validity and moderate reliability for assessing pain in mechanically ventilated pediatric patients under light to moderate sedation. However, these initial findings should be interpreted with caution, and its clinical application requires careful consideration of two major factors. First, the BPS exhibits a pronounced floor effect under deep sedation, indicating that low scores may reflect sedation-induced masking rather than true analgesia. Second, the variability in the correlations among observers suggests that adequate training and familiarity with the scale are essential for consistent pain assessment. Although the BPS is a useful objective alternative tool, our findings emphasize the need for adequate training, careful interpretation of scores in deeply sedated children, and the potential development of a pediatric-specific version of the BPS. Future research should evaluate the feasibility, observer burden, and interdisciplinary utility of the BPS compared to existing tools. Ultimately, while implementing the BPS as a common language across multidisciplinary teams could facilitate smoother communication in mixed adult–pediatric intensive care settings, these single-center results must be rigorously validated through future large-scale, multi-center prospective studies.

## Figures and Tables

**Table 1:** Characteristics of study patients in observations.

	Observations: 70
Male, n	50 (71.4)
Age, Years	1.7 ± 4.6
Age, Months	1 [0–8]
Age Category, n	
Neonate	21 (30.0)
Neonate–Under 1 year old	32 (45.6)
1–2 years old	7 (10.0)
3–5 years old	2 (2.9)
6–12 years old	2 (2.9)
13–18 years old	6 (8.6)
Body weight, kg	4.4 [3.1–5.7]
Classification, n	
Cardiovascular disease	51 (72.9)
Respiratory disease	9 (12.9)
Infection	6 (8.6)
Cerebrovascular disease	4 (5.6)
pSOFA score	6.8 ± 2.8
Sedation use, n	
Fentanyl	63 (90.0)
Midazolam	58 (82.9)
Dexmedetomidine	40 (57.1)
Deep sedation ^a^, n	48 (68.6)
Procedures ^b^, n	15 (21.4)

Abbreviations: pSOFA, Pediatric Sequential Organ Failure Assessment. Notes: Data are shown as mean ± standard deviation, median [IQR], or number (%). ^a^: Deep sedation was defined as a median Richmond Agitation–Sedation Scale (RASS) score of −4 or −5 across the three observers during the observation. ^b^: During observation, individuals underwent procedures causing pain (such as vital sign measurement, intravenous line placement, or suctioning).

**Table 2:** Measures of agreement between the observers: ICC and weighted κ.

	ICC (95% CI)	*p*	Weighted κ (95% CI)	*p*
Observations: 70				
All observations	0.66 (0.55–0.76)	<0.01	0.53 (0.39–0.66)	<0.01
Observers A–B	0.69 (0.55–0.80)	<0.01	0.69 (0.50–0.90)	<0.01
Observers B–C	0.72 (0.58–0.82)	<0.01	0.71 (0.48–0.86)	<0.01
Observers C–A	0.60 (0.42–0.73)	<0.01	0.63 (0.28–0.81)	<0.01
Compliance with ventilation				
All observations	-	-	0.53 (0.41–0.65)	<0.01
Observers A–B	-	-	0.65 (0.51–0.79)	<0.01
Observers B–C	-	-	0.68 (0.48–0.85)	<0.01
Observers C–A	-	-	0.51 (0.23–0.68)	<0.01
Movements of upper limbs				
All observations	-	-	0.08 (−0.03–0.24)	0.174
Observers A–B	-	-	0.49 (0.00–0.80)	<0.01
Observers B–C	-	-	0.00 (0.00–0.00) ^a^	1.00
Observers C–A	-	-	0.00 (0.00–0.00) ^a^	1.00
Facial expression				
All observations	-	-	0.38 (−0.03–0.69) ^b^	<0.01
Observers A–B	-	-	0.38 (−0.04–1.00) ^b^	<0.01
Observers B–C	-	-	0.36 (−0.05–0.74) ^b^	<0.01
Observers C–A	-	-	0.42 (0.00–0.79) ^b^	<0.01

Abbreviations: ICC—intraclass correlation coefficient. Notes: We calculated the ICC and weighted κ for the entire observer group (A, B, and C), and for each combination within it. For weighted κ, we calculated it not only for the entire scale but also for each subscale (compliance with ventilation, movements of upper limbs, and facial expression). ^a^: Weighted κ values of 0 occurred due to the “kappa paradox”, where observers demonstrated high agreement on a single rating (due to the pronounced floor effect from deep sedation), resulting in zero variance in the marginal totals. ^b^: Negative lower bounds of the 95% CIs occurred because the point estimates were close to zero, causing the asymptotic standard error approximation to extend below zero.

## Data Availability

Due to the ongoing sub-analysis of the data collected for this study, the datasets are not currently publicly available. Access to the data may be granted by the corresponding author upon receipt of a justified request. Researchers seeking to use these data for subsequent investigations are invited to contact Mr. Mitsuki Ikeda, the co-corresponding author, to discuss data accessibility.

## Supplementary Materials

The following supporting information can be downloaded at: https://www.mdpi.com/article/10.3390/children13060719/s1, Figure S1: Box plots for the association between BPS score and the presence of painful procedures; Figure S2: Box plots for the association between BPS score and deep sedation (RASS score < −3); Figure S3: Frequency distribution of pain assessment scores by observer; Figure S4: Scatter plots for Spearman rho correlation between BPS, FLACC and COMFORT-B scores by observer experience; Table S1: Characteristics of study patients at PICU admission; Table S2: BPS scores and procedure (mild to moderate sedation: RASS score ≥ −3).
