# Supplementary material for: Applicability, Validity, and Reliability of the Japanese Version of the Behavioral Pain Scale for Critically Ill Mechanically Ventilated Pediatric Patients: A Prospective Cross-Sectional Observational Study
Source: Children (Basel). 2026 May 22;13(6):719. doi: 10.3390/children13060719 (PMC13297250; doi:10.3390/children13060719)
Supplement: Supplementary file 1 [file children-13-00719-s001.zip › children-4293967-supplementary.pdf]

Table S1: Characteristics of Study Patients in PICU admission

	n = 37
Male, n	26 (70.3)
Age, Years	1.4 ± 3.9
Age, Months	1 [0–7]
Age Category, n	
Neonate	12 (32.4)
Neonate— Under 1 year old	17 (45.9)
1—2 years old	2 (5.4)
3—5 years old	2 (5.4)
6—12 years old	2 (5.4)
13—18 years old	2 (5.4)
Classification, n	
Cardiovascular disease	30 (81.1)
Respiratory disease	2 (5.4)
Infection disease	3 (8.1)
Cerebrovascular disease	2 (5.4)
Admission route, n	
Scheduled Surgery	24 (64.9)
Emergency Room	5 (13.5)
Non-Scheduled Surgery from in-hospital	4 (10.8)
NICU	2 (5.4)
Non-Scheduled Surgery from PICU	1 (2.7)
General ward	1 (2.7)
Length of stay, days	19 [12–32]
Length of mechanical ventilation, days	12 [5–27]
Developmental delay [a], n	5 (13.5)
Trisomy [b], n	6 (16.2)
PRISMIII score	18.2 ± 6.6

Abbreviations: NICU: Neonatal Intensive Care Unit; PICU: Pediatric Intensive Care Unit; PRISMIII: Pediatric Risk of Mortality III

Notes: Data are shown as mean ± standard deviation, median [IQR], or number (%).

a: Individuals diagnosed with developmental disorders or developmental delays in electronic medical records were categorized as having developmental delay.

b: Individuals diagnosed with trisomy 13, 18, or 21 in the electronic medical records were classified as having trisomy.

Table S2: BPS scores and Procedure (Mild to Moderate Sedation: RASS ≥ -3)

Observer	n<br>No Procedure	BPS<br>No Procedure	n<br>Procedure	BPS<br>Procedure	*p*	Effect Size (r)
A	46	3 [3–3]	11	3 [3–4]	0.35	0.13
B	46	3 [4–4]	11	3 [3–4]	0.62	0.07
C	46	3 [3–3]	11	3 [3–4.5]	0.08	0.24

Abbreviations: BPS: Behavioral Pain Scale

Notes: Data for BPS scores are expressed as median [interquartile range]. *Mild to moderate sedation* was defined as a Richmond Agitation-Sedation Scale (RASS) score of -3 or higher. *Procedure* indicates that pain-inducing routine procedures (e.g., vital sign measurement, intravenous line placement, or suctioning) were performed during the observation. The *p*-values and effect sizes (*r*) were calculated using the Mann-Whitney U test.

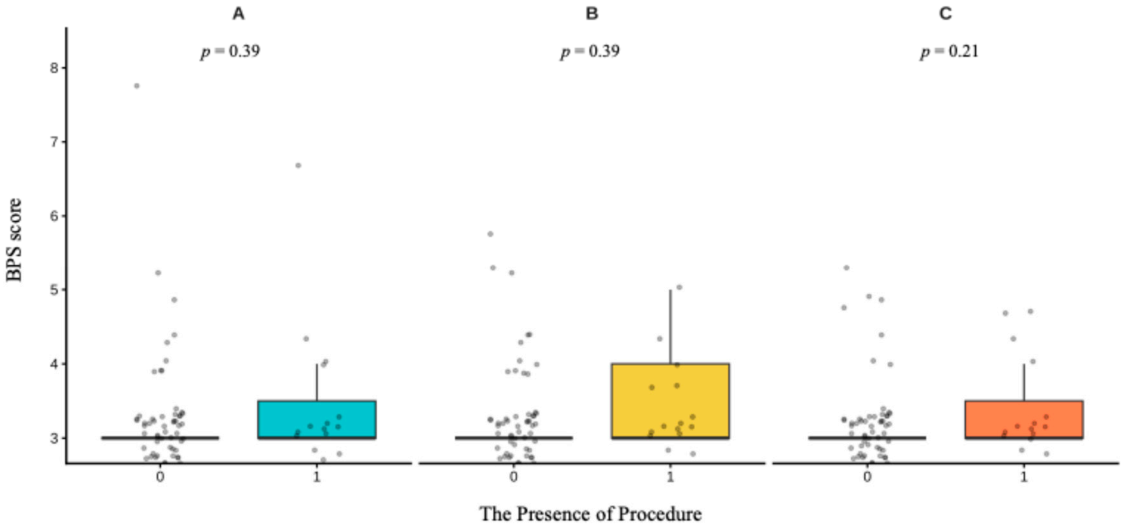

Figure S1: Box plots for the association between BPS and the presence of Painful Procedure.

Abbreviations: BPS: Behavioral Pain Scale

Notes: The Presence of Procedure show that individuals with a procedures causing pain (such as vital sign measurement, IV line placement, or suctioning) were performed during observation.

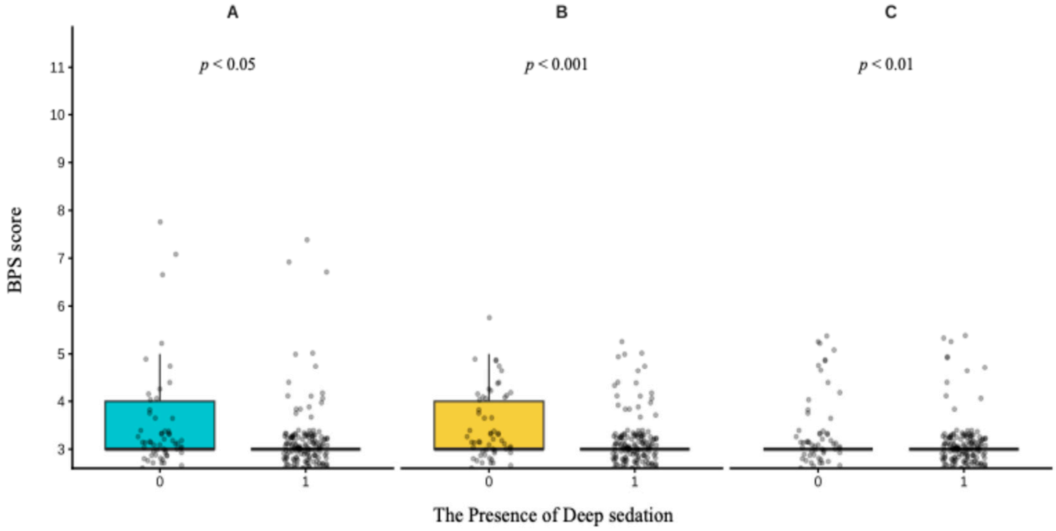

Figure S2: Box plots for the association between BPS and Deep Sedation: RASS ≤ −4.

Abbreviations: BPS: Behavioral Pain Scale.

Notes: Deep sedation was defined as a median Richmond Agitation Sedation Scale (RASS) score of ≤ -4 across the three observers.

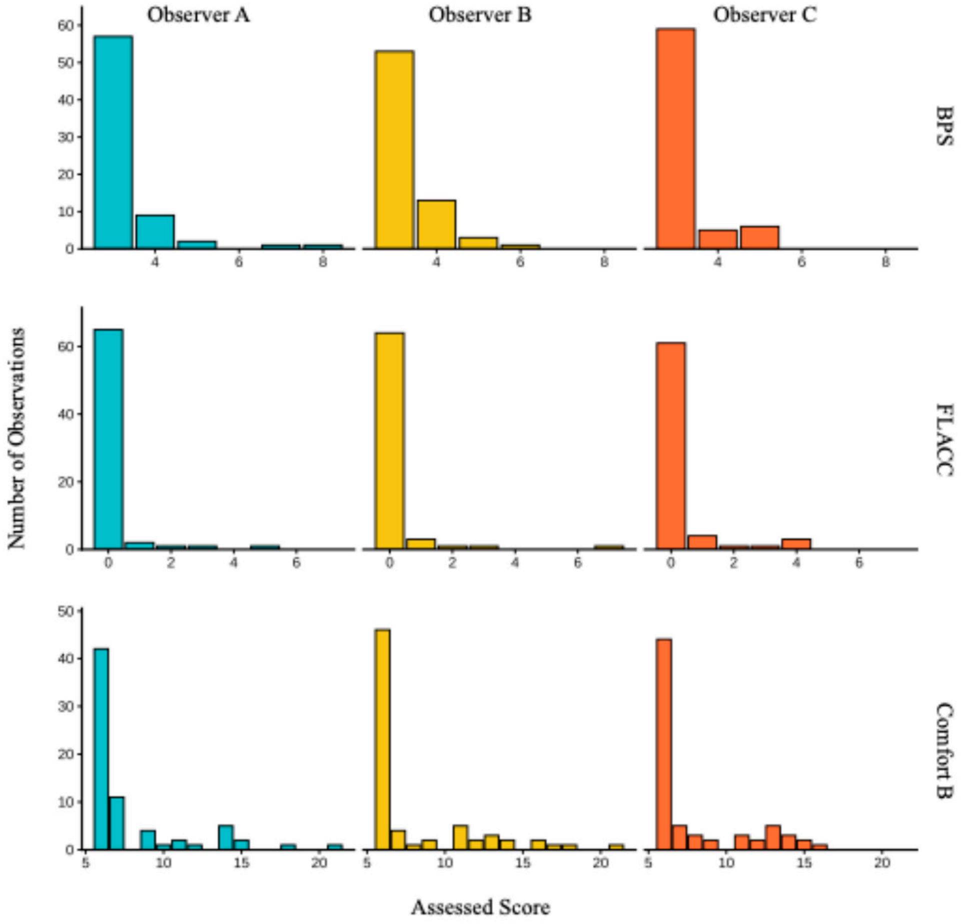

Figure S3: Frequency Distribution of Pain Assessment Scores by Observer

Abbreviations: BPS: Behavioral Pain Scale; FLACC: Face, Legs, Activity, Cry, Consolability.
Notes: Data are expressed as the absolute number of observations. The possible score ranges for each pain assessment tool are as follows: BPS, 3 to 12; FLACC, 0 to 10; and COMFORT-B, 6 to 30.

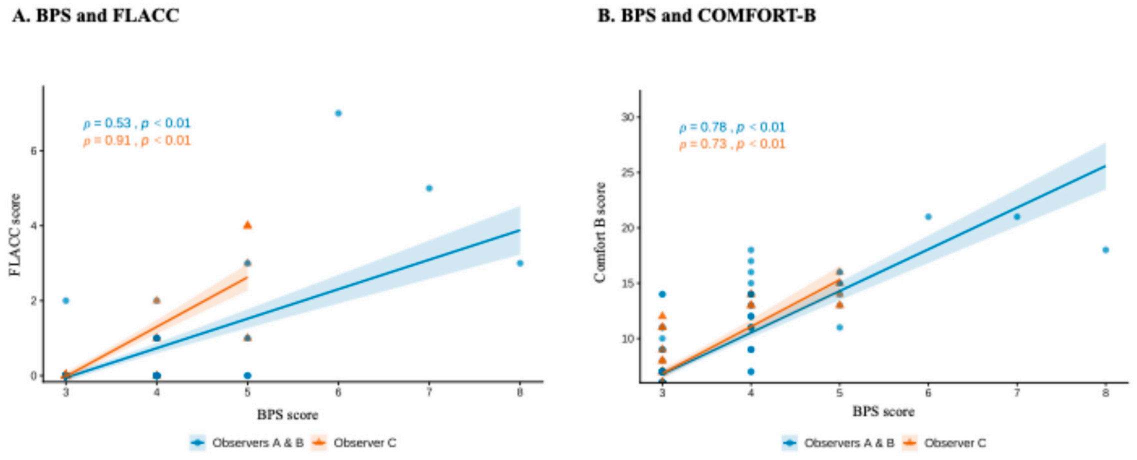

Figure S4: Scatter Plots for Spearman rho correlation between BPS, FLACC and COMFORT-B by Observer Experience

Abbreviations: BPS: Behavioral Pain Scale; COMFORT-B: COMFORT Behavior Scale; FLACC: Face, Legs, Activity, Cry and Consolability Scale.

Notes: For the Spearman's rho correlation coefficients, we plotted scatter diagrams grouped by the observers' prior experience with the BPS. Observers A and B represent nurses with limited prior experience with the BPS, whereas Observer C represents a nurse with extensive adult ICU experience and high familiarity with the BPS, in addition to PICU experience.
